# Enhancing research for undergraduates through a nanotechnology training program that utilizes analytical and bioanalytical tools

**DOI:** 10.1007/s00216-018-1274-5

**Published:** 2018-08-18

**Authors:** Lisa A. Holland, Jeffrey S. Carver, Lindsay M. Veltri, Rachel J. Henderson, Kimberly D. Quedado

**Affiliations:** 1 C. Eugene Bennett Department of Chemistry, West Virginia University, 217 Clark Hall, Morgantown, WV 26506 USA; 2 Curriculum and Instruction/Literacy Studies, West Virginia University, 602 Allen Hall, Morgantown, WV 26506 USA; 3 Department of Physics and Astronomy, Michigan State University, 567 Wilson Rd, East Lansing, MI 48824 USA; 4 Office of Undergraduate Research, Honors College, West Virginia University, 250 Second Street, Morgantown, WV 26506 USA

**Keywords:** Nanotechnology, Undergraduate research, REU, Training program

## Abstract

Nanotechnology is a broad field combining traditional scientific disciplines; however, analytical chemistry plays an important role in material design, synthesis, characterization, and application. This article emphasizes the uniqueness of nanotechnology and the importance of providing high-quality undergraduate research experiences to both attract and retain talented individuals to the field of nanotechnology. In response to this need to develop a strong and sustainable nanotechnology work force, strategies to create authentic research experiences are considered within the framework of an interdisciplinary nanotechnology environment at West Virginia University. The program, named NanoSAFE Research Experiences for Undergraduates (REU), embeds students in different departments at West Virginia University and in research laboratories within the National Institute of Occupational Safety and Health. A large number of participants have little or no prior research experience and a strong effort is made to recruit applicants from under-represented populations. Components designed to foster research proficiency include frequent reporting, a strong peer-network, and training for secondary mentors. Evidence, which includes student publications and assessment findings demonstrating self-efficacy, is discussed to substantiate the viability of the strategies used in the 2016–2018 program.

**Electronic supplementary material:**

The online version of this article (10.1007/s00216-018-1274-5) contains supplementary material, which is available to authorized users.

**Graphical abstract:**
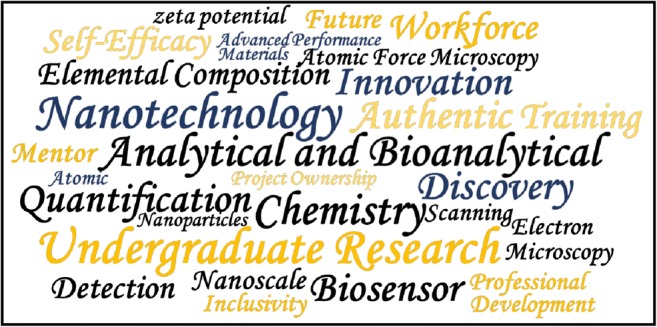
ᅟ

## Introduction

Nanotechnology supports the creation of safe and sustainable advanced materials for the next generation of manufacturing and technology [1, 2], with the R&D expenditures in the business sector for 2014 estimated at 17.6 billion dollars for the USA [3]. The field of nanotechnology is broad and is founded on the convergence of traditional disciplines to create, study, and apply materials at the nanoscale. Although the science, technology, engineering, and math (STEM) disciplines that contribute to nanotechnology are diverse, analytical and bioanalytical technologies are fundamental to answer critical research questions. Measurements of chemical and physical properties are integrated throughout this process. Examples include qualitative and quantitative analyses of elemental composition, assessment of molecular adsorption, measurement of binding or affinity, and even evaluation of size.

Advancing nanotechnology requires new strategies to recruit and train talented researchers in this field [4]. Diversity in the researchers engaged in nanotechnology is appealing because diverse teams arrive at more innovative solutions to challenges [5]. While different approaches are documented to foster diversity [6–8], undergraduate research experiences are identified as one component of a multifaceted approach to increase diversity [9, 10] by recruiting and retaining highly talented STEM researchers from under-represented populations (UREP). In nanotechnology, females are considered UREPS [11]. Training environments that create multidisciplinary or interdisciplinary environments for early research experiences, such as National Science Foundation (NSF) Research Experiences for Undergraduates (REUs), are fundamental to prepare the next generation of scientists and engineers. The benefits of research experiences, which include fostering critical thinking as well as increasing retention in STEM fields, have been articulated previously [12–16]. REU programs, which are funded through the NSF to enhance undergraduate education [17], are one mechanism to advance STEM education by providing research opportunities that would otherwise be unavailable to some students. These programs deliver competitive research projects to undergraduates, typically in summer, providing stipend and subsistence. An REU program is an ideal test-bed to implement and evaluate inclusive strategies to rapidly train students in research because the transformative research experiences must be compressed into a 10-week timeline. The impact of nanotechnology in undergraduate research is apparent as 15 unique NSF-REU sites have nanotechnology as the programmatic theme [18]. Nanotechnology research flourishes in the presence of a broad range of disciplines, expertise, and instrumentation.

The nanotechnology REU site at WVU, informally referred to as WVU NanoSAFE (Nanotechnology Sensing Advances in Field and Environment) REU, is an authentic training network deeply embedded in a sustainable nanotechnology program. This is facilitated by high activity in the field of nanoscale science and engineering [19] and access to an extensive set of analytical and bioanalytical instrumentation through faculty research labs as well as a shared research facility at the University that provides expensive and sophisticated equipment critical to supporting nanotechnology research. The goal of the NanoSAFE REU program is to provide rich nanotechnology research experiences that include UREPS. Over the course of 3-years (2016–2018), the NanoSAFE REU program admitted 36 students. Females compose 67% of the cohort and 28% of the cohort belong to other diversity groups (Hispanic or Latino, African American, Native American). A majority of the cohort is Appalachian (69%) and 25% of the cohort self-identified as a first-generation college student. Twenty faculty (13 male, 7 female) serve as research mentors to participants. Four of the faculty mentors hold appointments at both WVU and the National Institute of Occupational Safety and Health (NIOSH) and are involved in establishing safety guidelines for workers in the rapidly expanding sector of nanotechnology manufacturing.

## Research training at the WVU site: multifunctional nanomaterials

**Fig. 1:**
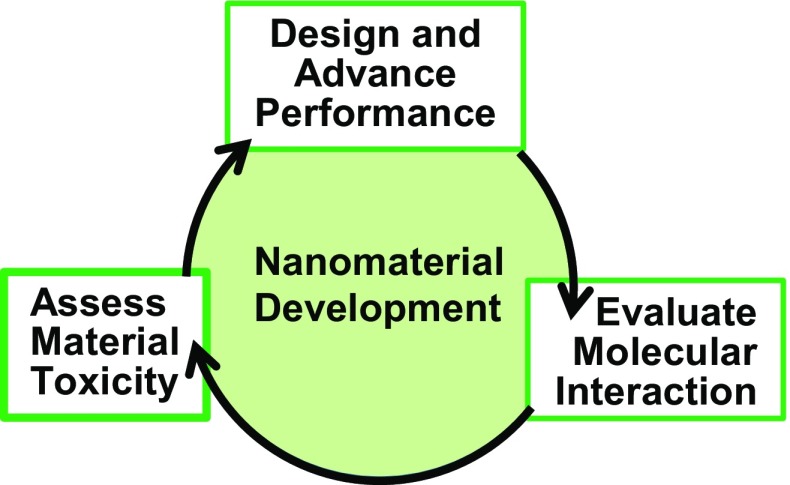
The integrated nature of nanotechnology research requires an iterative disciplinary approach not often addressed in traditional educational settings

**Fig. 2:**
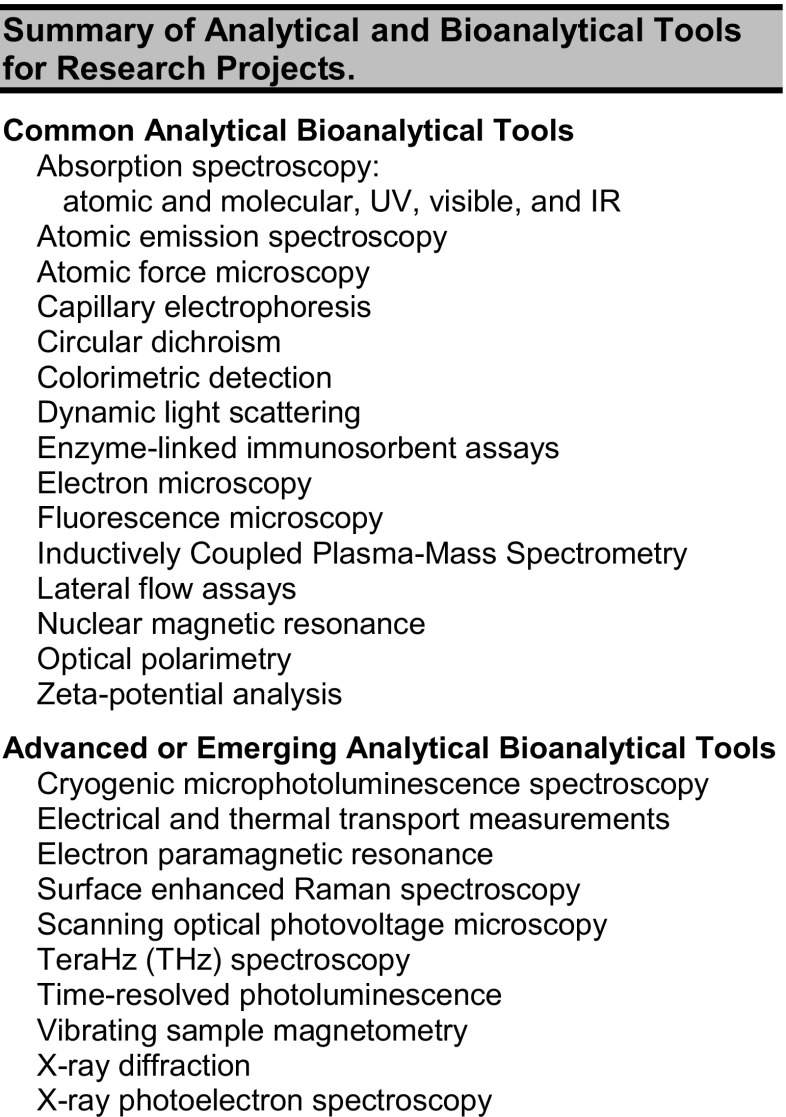
Summary of analytical and bioanalytical tools for research projects

Research in nanotechnology is based on the convergence of multiple disciplines. Advanced performance nanomaterials are designed, characterized, and refined. As summarized in Fig. 1, these activities foster an iterative approach to fabricate, evaluate, and perform toxicity assessment necessary to realize high-performance nanomaterials that can safely support manufacturing processes. Although the research disciplines are diverse, each research project relies on analytical and bioanalytical technology. Researchers in the program employed a wide variety of instrumentation (see Fig. 2) to evaluate biomolecular interfaces, to confirm fabrication progress, and to assess the effect of nanomaterials both in vivo and in vitro. The challenges of nanotechnology research, which include unique material properties and stringency in size and elemental composition, have led to the recognition that scientists must adapt traditional tools or even create new tools in order to continue the pace of nanotechnology innovation and discovery well into the future [20]. A hallmark of the NanoSAFE REU program is student access to cutting-edge instrumentation either as a tool or as emerging analytical tools (see Fig. 2). This interdependence of nanotechnology and analytical bioanalytical chemistry creates a training environment in which students must learn basic principles of analyses and then harness them appropriately to design and test research hypotheses.

The educational goals of the NanoSAFE REU program are to help students who have limited access or engagement in authentic research experiences within the limited time-frame of an REU. The undergraduate participants enter with a diverse range of research experience, with many having little (11%) or no (44%) research experience prior to the program. The program also maintains an emphasis on inclusion of participants who are attending a primarily undergraduate institution (89%).

The WVU NanoSAFE REU site began in 2007 and has evolved with each 3-year life span of NSF support. In the prior 9 years of the program (2007–2009, Award #DMR-0647763; 2010–2012, Award #DMR-1004431; 2013–2015, Award #DMR-1262075), 96 participants were served and a total of 5 participants were co-authors on manuscripts [21–25]. The act of communicating research results through publications or presentations is one of the nine characteristics that define an authentic undergraduate research experience [26]. An aspiration of the current NanoSAFE REU program is to ensure that as many participants as possible engage in research to such an extent that they are co-authors on publications. New infrastructure, which includes frequent reporting, a strong peer-network, and training for secondary mentors, was implemented in the program in an effort to increase the research proficiency of the participants. With this increased focus on enhancing research proficiency, the 2016–2018 program has promising outcomes. Four of the 12 participants from the 2016 cohort became co-authors on research publications [27–30] and 5 of the 12 participants from the 2017 cohort have reported being included on manuscripts in preparation. Other outcomes of the current REU program are worth noting. In the 2016 WVU Summer Undergraduate Research Symposium, all participants were assigned to the same poster discipline (Nanoscience), with the outcome of one participant being awarded category winner and one being named runner up in the poster competition. In the 2017 WVU Summer Undergraduate Research Symposium, participants were assigned to four different judging disciplines (Biological Science, Engineering, Health Science, Nanoscience), with the outcome of one participant being awarded category winner and two participants being named runner ups in the poster competition. The program has already documented the awarding of an NSF Graduate Research Fellowship, which is a highly competitive and prestigious award.

## Program elements for professional development

**Fig. 3:**
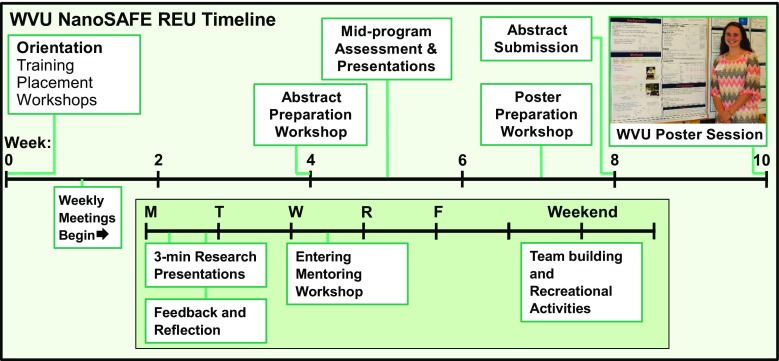
The research experience is intensive and culminates with poster presentations at the WVU Summer Undergraduate Research Symposium

The WVU NanoSAFE REU program focuses on developing the framework for students with different cultural experiences to succeed. Professional development is distributed throughout the 10-week period of intensive research with training elements that include communication workshops (e.g., creating abstracts and posters), mentor training, and team-building (see Fig. 3). Some of this infrastructure is provided through the WVU Office of Undergraduate Research, which seeks to sustain a vibrant summer undergraduate research environment through multiple research initiatives [31]. Recreational team-building exercises (ropes course, Escape Room, white-water rafting) are arranged on weekends to create a sense of community as a means to facilitate peer-mentoring. The role of secondary mentors in research training was supported in the NanoSAFE REU program and weekly training modeled after the Entering Mentoring seminar [32] was made available to graduate students who served as secondary mentors.

## Program elements for research proficiency

A priority of the program is to bring students up to speed quickly. The first week is particularly rigorous as students are taught many concepts fundamental to engage in research. This includes discussion on communicating the significance or innovation inherent in the research and the potential societal impact that makes the work valuable to pursue. The undergraduate researchers are also trained to differentiate scientific discovery from laboratory activity. This is to convince them that discovery is integral to an authentic research experience; whereas, activity stalls growth and is counterproductive to the intellectual investment associated with research independence. Individuals in the REU program tour research labs the first day, having already been informed of the projects through a one-page description and recorded presentation outlining the goals of each project and are placed in a research lab by the second day. Safety training, research expectations, and library skills are delivered at onset. At the close of the first week, each researcher prepares and delivers a 10-min description of his or her understanding of their summer research. This activity helps the REU participants to document a personal vision of the research project including each student’s perception of the significance, innovation, and fundamental principles that drive the scientific questions relevant to the project.

To help students comprehend their projects, research progress is documented weekly using structured presentations and weekly assessment (self- and peer-). These weekly reporting meetings are facilitated with web-conferencing because the different research labs within WVU and NIOSH are not adjacent to each other, although they are co-located within Morgantown. This eliminated the requirement for weekly travel and also provided a mechanism to record the weekly presentations. Weekly group meetings and review of recorded reports make this an iterative process where students become skilled at critiquing their own findings and those of their colleagues. The expectation is that the process enables the participant to draw conclusions independently with less intervention by the secondary mentor (e.g., graduate student) and primary mentor (i.e., the faculty advisor).

**Fig. 4:**
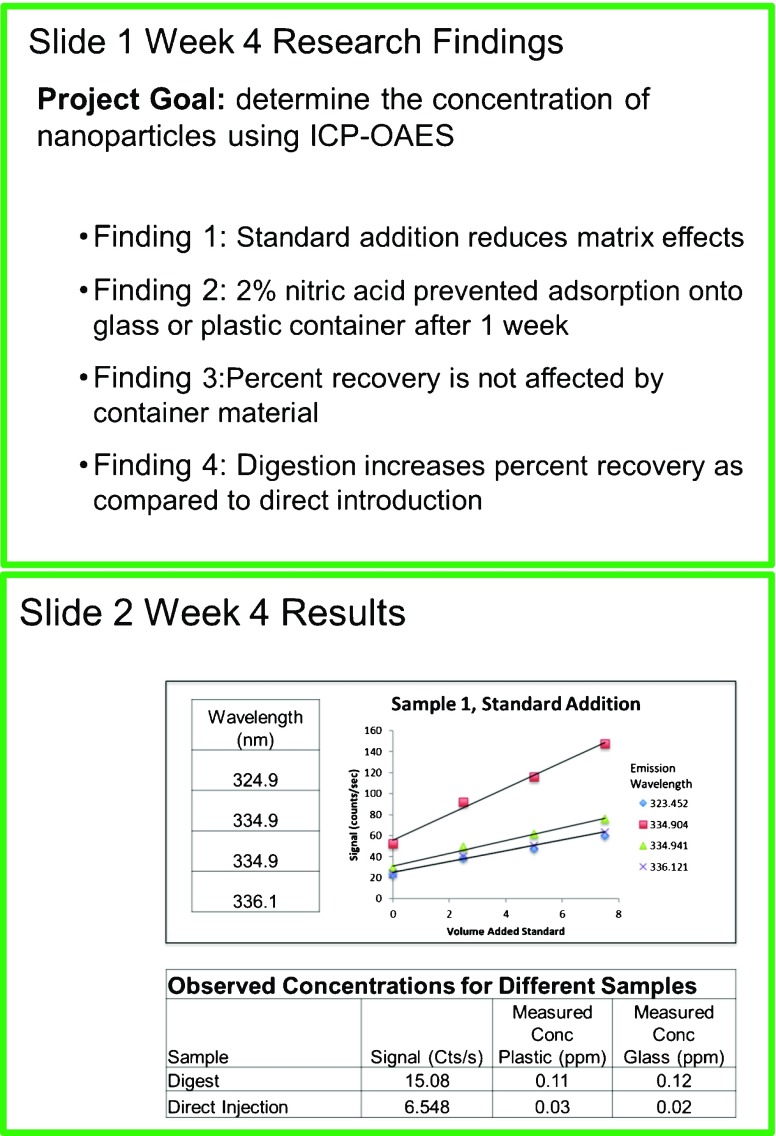
An example of a template for the weekly presentation slides. The purpose of the first slide is to specify project goals and summarize relevant discoveries made within the reporting time period, while the second slide presents the supporting data

A structured slide format is used (Fig. 4) to enable students to identify and effectively communicate only the most pertinent details of the research project. With only two PowerPoint slides, each student is allotted 3 min to communicate weekly goals and major discoveries aligned with the 10-week project (slide 1) and to describe how the hypothesized discovery is substantiated by data obtained during the week (slide 2). The template and short reporting time forces each participant to internally define features of a successful experiment for which the hypothesis test is designed appropriately. The figure elements created each week are continually refined and collected to form the body of results presented in the final poster. This helps students to build a library of data figures and to develop their verbal and visual presentation skills throughout the 10-week program, rather than at the end of the research experience.

## Assessment of the WVU NanoSAFE program

The achievements of the WVU NanoSAFE undergraduate participants warranted an examination of skills and knowledge in order to identify specific aspects of the program that contribute the most to these outcomes. Traditionally, REU programs are assessed using surveys to gain insight into student perception of progress. The Undergraduate Research Student Self-Assessment, more commonly recognized as URSSA, and Survey of Undergraduate Research Experiences, referred to as SURE, are publicly available tools to quantitatively evaluate undergraduate researchers [33, 34]. To provide insight into the potential of specific elements of program infrastructure to contribute to research proficiency, a combination of different quantitative and qualitative tools was administered. Students were given a survey reported previously [35, 36], of which many of the questions were aligned with those reported in the Survey of Undergraduate Research Experiences [37]. Students reported gains in their knowledge and skills between the onset and conclusion of the 10-week NanoSAFE REU program (see Table 1). The results of the survey indicate a high level of satisfaction and perceived gains in skills and knowledge associated with research proficiency.

**Table 1:** Perceived knowledge gains for NanoSAFE students in 2016 and 2017

Skill/Level/Knowledge in:	Pre-REU (*n* = 20)	Post-REU (*n* = 20)	Gain (*n* = 20)
Understanding scientific papers	3.33	4.50	1.17^c^
Using research equipment	3.25	4.65	1.40^c^
Formulating research hypotheses	3.00	4.40	1.40^c^
Developing a research project	2.55	4.25	1.70^c^
Conducting a research project	2.95	4.95	2.00^c^
Analyzing data	3.45	4.75	1.30^c^
Giving feedback to a peer	3.65	4.45	0.80^a^
Receiving feedback	4.15	4.80	0.65^a^
Presenting information	3.60	4.55	0.95^c^
Articulating questions	3.50	4.40	0.90^b^
Dealing with setbacks	3.60	4.80	1.20^c^
Working independently on research	3.15	4.60	1.45^c^
Working collaboratively with others	4.20	5.00	0.80^b^
Conversing with research faculty	4.05	4.75	0.70^b^
Research skills in general	3.40	4.70	1.30^c^
The nature of science and research	3.73	4.55	0.82^c^
The nature of the job as a researcher	3.20	4.75	1.55^c^
Career paths of science faculty	3.40	4.60	1.20^b^
What graduate school is like	2.30	4.95	2.65^c^
Career options in the sciences	3.80	4.80	1.00^b^

A perceived knowledge pre-survey based on a 6-point Likert scale was administered during the first day of the program (pre) and again on the last day of the program (post). For each participant and question, perceived knowledge gains were calculated and then averaged*.* All gains are statistically significant are denoted with an asterisk. The value for ρ is calculated using a paired *t* test to analyze significant differences between the pre and post assessments. Statistical significance is denoted with a superscript: “a” denotes *p* < 0.05, “b” denotes *p* < 0.01, and “c” denotes *p* < 0.001

Peer-assessment of posters was conducted to determine how students perceive their performance relative to other undergraduates motivated to succeed in research. The undergraduates in the 2017 NanoSAFE REU cohort were asked to evaluate posters from undergraduate researchers who participated in the WVU Summer Undergraduate Research Symposium. All 12 participants in the 2017 cohort were assigned to 1 of 3 groups based on the similarity of the disciplinary session in which the poster of the participant was entered. Each group evaluated eight elements (see Table S1 in the Electronic Supplementary Material, ESM) of three posters in the same or in a similar poster session discipline by NanoSAFE REU colleagues or of three other researchers outside of the REU program. The results, summarized in Table S2 in the ESM, reveal that the NanoREU students viewed their performance as equivalent or better than those of other undergraduate researchers with similar or more research experience. These preliminary findings demonstrate that at the conclusion of the program, the NanoSAFE REU students had confidence in their performance relative to their broader peers also engaged in research. The findings from quantitative surveys provide evidence of research self-efficacy as students viewed themselves and their peers as competent researchers.

Evaluations of research accomplishments were also performed based on weekly reporting held using web-conferencing. These quantitative assessments were in the form of surveys containing three questions (see Table S3 in the ESM) with Likert responses (strongly disagree, disagree, neither agree/disagree, agree, strongly agree) to assess communication (question 1), discovery (question 2), and the ability to defend experimental findings (question 3). The scores related to each presentation were averaged, and self-evaluations excluded. The results from peer assessment do not reveal significant gains because students rated themselves highly from program onset. Although gains were not significant, the surveys revealed that students in the cohort were equally satisfied with the performance of their colleagues throughout the program, rating all categories high from onset and throughout the duration of the program (see Tables S4, S5, S6 in the ESM).

Narrated presentations were also evaluated for project ownership using qualitative assessment. This concept of project ownership provides more insight about each student’s perception of their mastery as well as investment in undergraduate research. A qualitative analysis of language used during interviews revealed that undergraduate research experiences are more effective than teaching laboratories at fostering project ownership, that project ownership contributes to persistence in science, and that students express different degrees of project ownership [38]. The narrated presentations produced by the 2017 cohort of the NanoSAFE REU program were transcribed word for word and each student researcher’s transcripts were connected together in consecutive order. Transcripts were analyzed using a closed coding system to categorize the data, looking specifically for language indicating ownership of the presentation, research ideas, research tasks, and research findings in order to document the degree to which each participant took individual ownership of the project, goals, or findings. These transcripts were coded and then grouped based on emerging trends in language usage.

The study involved qualitative analysis that focused on the qualitative manner in which participants used language. As this was not based on statistical data, no statistical significance can be calculated. However, the qualitative assessment of the narrated presentations, described in detail in the ESM, brought several points to light regarding project ownership. A principle finding is that each of the 2017 REU participants demonstrate ownership of their presentations, with all referring to themselves (e.g., “as I’ve shown here,” “as I’ve stated previously,” “my graph here shows…”) when speaking about something specifically related to the presentation. This confirms that each student created his or her own slides. In maintaining ownership of their presentations, every undergraduate researcher exhibits a degree of independence regarding the experience.

Results related to owning the research project are also informative. During the 10-week period of research, of the 12 students that composed the cohort, 5 did not demonstrate any clear progression in ownership, while 3 began the program with a strong perception of independence that did not falter. Only 4 of the 12 students either progressed or transitioned between individual and group ownership. There was no correlation between ownership of the research project and gender, ethnicity, or prior research experience. These results reflect the unique requirements of both research training and of nanotechnology. First, engaging in research training requires participating in an authentic research question beyond the boundary of what is known in the field. Entering researchers must experience research guided through mentoring in order to advance to a level that allows them to develop and design their own informed questions that push the boundaries of science. Second, nanotechnology is founded at the cross-section of different disciplines. As a result, entering researchers are often embedded in research projects that require a broad range of disciplinary expertise. Success in this type of setting requires researchers to exist within a duality of individual versus team-based research. This aspect of nanotechnology is an ideal environment for research training because it provides opportunities to exercise independent research within the framework of a scientific team providing mentorship and support.

## Program reflection

Several features of the program were found to work well, including the use of web-conferencing to eliminate geographical barriers, a specific group meeting format to stream-line group reporting time and accountability for research progress, and the development of a strong feedback network to support participant development. In spite of these findings, further improvements can be made. The biggest challenge is related to developing assessment that can provide insight into the progress of individual students. With personalized assessment, interventions could be tailored that are specific to the needs of a particular student.

A variety of different assessment strategies are accepted or emerging for REU program evaluation [26]. Although it is widely used, self-assessment presents difficulties when the subject lacks a mature understanding of success or of progress. Strategies used in conjunction with surveys have also been described including conversation [39] or analyses of writing samples [40]. Entering researchers may not know how to evaluate themselves simply because they are too inexperienced to be aware of their shortcomings [41]. They also may possess different biases that influence the accuracy and objectivity of the results [42]. Alternative strategies, such as asking experienced researchers to evaluate entering researchers, pose other problems. Established researchers develop different disciplinary standards, observe different cultural practices, and possess unique biases as a result of personal experiences which collectively create a range of evaluation results. These issues may be alleviated, but not eliminated, for many forms of evaluation if an effective rubric is provided to assist in assessment.

Broad goals, such as research proficiency, are known to require lengthy periods of time as observed in average time-to-degree in graduate research training of 6–7 years for physical science, engineering, and math [43]. The process of becoming proficient in research is complex and advancement in a specific area may be predicated on developing a set of pre-requisite skills [44]. If a combination of new or improved knowledge and/or skills is required for proficiency, failure to survey critical elements leading to progression may obscure evidence of success. Even more daunting, if a combination of gains with a wide dynamic range is needed and some elements require only incremental improvements, this makes obtaining evidence of skills that lead to success more elusive.

A continuum of evaluation methods is required to address the diversity of approaches to deliver high-quality research experiences that are uniquely developed to meet specific needs for undergraduate training in the STEM community. In support of this endeavor, the NanoSAFE REU program continues to evaluate and evolve the training infrastructure. Combining qualitative assessment with quantitative surveys will shed light on the unique path of growth in individual researchers. Future studies also include less structured means of assessment such as journaling and including more opportunities for personal reflection.

## Conclusions and future directions

The WVU NanoSAFE REU program is based on diverse nanotechnology research that is aligned with the same goal to advance society through a new class of advanced performance materials that are safe and sustainable. The program leverages strong intellectual and instrumental resources across the Eberly College of Arts and Sciences, the Benjamin M. Statler College of Engineering and Mineral Resources, the Robert C. Byrd Health Sciences Center, and NIOSH. Several strategies including surveys, qualitative assessment, and tracking of research metrics (publications, presentations, awards) have provided formative assessment of this program to address the current needs of students. Although the present findings demonstrate the achievements of this REU, a deeper understanding of student development will add new perspective to an active STEM education community that seeks to guide educators to advance STEM training and enhance the body of STEM researchers and STEM research. Continued observation of narrated presentations captured throughout the program offers a means to review metrics of progression (e.g., psychological ownership, self-efficacy, independence, enthusiasm). Quantitative surveys should expand the current understanding of how different skill sets contribute to progression (e.g., problem solving, literature skills, interpretation, or critical thinking).

The perspective presented in this paper outlines one example of educational practices to attract individuals to the field of nanotechnology and advance their professional trajectory. This program is specifically designed for students familiar with predominantly undergraduate institutions in Appalachia. In addition to serving as a bridge for students to transition from training programs at small schools in Appalachia, an advantageous feature of nanotechnology training at WVU is that training in a single institutional setting still enables undergraduate researchers to participate in projects that span academic departments in engineering, health sciences, and basic sciences, as well as government laboratories. New paradigms in educational training programs are also necessary to realize the benefits of an inclusive training environment and the need to adapt and potentially revise educational strategies in order to foster greater achievements by individuals with different cultural experiences and expectations.

Participants in the WVU NanoSAFE REU program have expressed satisfaction with nanotechnology research experiences at WVU for 12 years; however, the program is continuously revised to improve the training experience and outcome. This also allows the faculty involved in the WVU NanoREU to change the program on-demand to better serve participants. While nanotechnology is changing, the complex and dynamic experiences of people entering workforce training in nanotechnology requires continual assessment of the effectiveness of training strategies. Approaches to educational assessment continue to evolve as researchers uncover the processes associated with attaining skills and knowledge. This in turn will increase and enhance the collective research proficiency in fields such as nanotechnology that advance and innovate society through better products, improved manufacturing, and enabling tools for society.

## Electronic supplementary material

ESM 1 (PDF 451 kb)
