# Supplementary material for: Enhancing research for undergraduates through a nanotechnology training program that utilizes analytical and bioanalytical tools
Source: Anal Bioanal Chem. 2018 Aug 18;410(24):6041–50. doi: 10.1007/s00216-018-1274-5 (PMC6132685; doi:10.1007/s00216-018-1274-5)
Supplement: Supplementary file 1 — (PDF 451 kb) [file 216_2018_1274_MOESM1_ESM.pdf]

Analytical and Bioanalytical Chemistry

Electronic Supplementary Material

Enhancing research for undergraduates through a nanotechnology training program that utilizes analytical and bioanalytical tools

Lisa A. Holland, Jeffrey S. Carver, Lindsay M. Veltri, Rachel J. Henderson, Kimberly D. Quedado

Abstract. The following is the supplementary data related to this article: Tables summarizing survey questions, results of REU self-assessment, results of REU peer-assessment, and detailed discussion of the results of the qualitative analysis.

Table S1. Questions Used for Poster Surveys[a]

(1) Abstract: Summarized the research and results by only providing relevant information and simple language.
(2) Introduction: Provided relevant information toward the overall objective of the project in an organized fashion.
(3) Methods: Presented experiments that are explained and connected to the overall objective of the project
(4) Results: Presented in a figure or diagram and explicitly show how they support the overall objective of the project
(5) Conclusion: Stated clearly, relevant and connected to the results
(6) Significance: Presented the importance of the research project
(7) Broader Impact: Presented “the potential to benefit society and contribute to the achievement of specific, desired societal outcomes”[b]
(8) Innovation: Presented the transformation between scientific knowledge and the economic enterprise
(9) Experience: Time spent in research

[a]Questions 1-7 are adapted from: Branchaw J, Pfund C, Rediske R (2010) Entering Research : A Facilitator's Manual : Workshops for Students Beginning Research in Science, Chapter 21 Making Effective Scientific Presentations see poster evaluation rubric, page 209. W.h. Freeman Scientific Teaching Series. W.H. Freeman, New York, NY,
[b]National Science Foundation, Proposal & Award Policies & Procedures Guide, Chapter III - NSF Proposal Processing and Review, available at https://www.nsf.gov/pubs/policydocs/pappg18_1/pappg_3.jsp#IIIA, accessed May 1, 2018

Table S2. Summary of Poster Evaluations by 2017 NanoSAFE REU Participants

	Group 1		Group 2		Group 3	
	control	NanoREU	control	NanoREU	control	NanoREU
abstract	3.0±0.9	4.9±0.2	3.9±0.1	3.9±0.4	3.3±0.4	3.6±0.6
introduction	4.5±0.4	4.7±0.0	4.3±0.1	4.6±0.2	4.3±0.3	4.5±0.1
methods	4.3±0.3	4.7±0.3	4.2±0.1	4.5±0.2	3.5±0.9	4.8±0.3
results	3.9±0.5	4.9±0.2	4.1±0.5	4.4±0.4	4.1±0.3	4.5±0.2
conclusions	4.3±0.4	4.9±0.2	4.1±0.1	4.4±0.3	4.3±0.4	4.5±0.3
significance	4.2±0.2	4.4±0.7	4.2±0.4	4.3±0.5	3.9±0.7	4.1±0.6
impact	3.9±0.5	4.7±0.3	4.2±0.1	4.0±0.3	3.8±0.6	4.0±0.5
innovation	3.7±0.3	4.7±0.3	4.1±0.3	3.9±0.3	3.5±0.0	3.8±0.3
experience	ND	weeks	>weeks	>weeks	>weeks	weeks

Eleven NanoSAFE REU participants in the 2017 cohort completed surveys about posters. Each group was composed of 4 NanoSAFE REU students in the same or similar poster session disciplines; however, only 3 students were responsive to the survey exercise in group 1. Individuals in the group evaluated the posters presented by NanoSAFE REU colleagues in that group and 3 posters of researchers not associated with the NanoSAFE REU program, but in the same poster discipline. Note that some REU students in Groups 2 and 3 also included self-evaluations. If duration of research was not determined it is labeled ND.

Table S3. Questions Used for Narrated Presentations[a]

(1) Specific objectives that contribute to the overall research project goal were effectively communicated.
(2) The stated findings were important experimental discoveries as opposed to tasks performed in a scientific experiment
(3) The experimental evidence presented effectively supported the stated scientific findings

[a]A 5-point Likert scale accompanies each question

Table S4. REU Assessment of REU Peers[a]
Question 1: research goals communicated

	Week 2			Week 10		
Subject	Ave		SD	Ave	±	SD
1	4.5	±	0.5	4.6	±	0.5
2	4.4	±	0.7	4.6	±	0.5
3	$4._{1}$	±	$1._{0}$	4.5	±	0.5
4	4.5	±	0.5	4.5	±	0.5
5	4.1	±	0.9	4.5	±	0.5
6	4.4	±	0.7	4.5	±	0.5
7	4.4	±	0.7	4.4	±	0.7
8	$4._{1}$	±	$1._{0}$	4.0	±	0.8
9	4.5	±	0.5	4.5	±	0.5
10	4.5	±	0.5	4.4	±	0.5
11	$3._{8}$	±	$1._{3}$	4.4	±	0.7
12	4.5	±	0.5	4.6	±	0.5

[a]Values are the average of 5-point Likert scale responses to questions in Table S-2 of 11 REU peers (self evaluations were excluded)

**Table S5.** REU Assessment or REU Peers[a] Question 2: discovery-based findings reported						
	Week 2			Week 10		
Subject	Ave		SD	Ave	±	SD
1	4.5	±	0.7	4.5	±	0.5
2	$3._{8}$	±	$1._{1}$	4.4	±	0.8
3	$3._{9}$	±	$1._{0}$	4.2	±	0.9
4	4.3	±	0.8	4.4	±	0.7
5	4.0	±	0.9	4.2	±	0.9
6	4.1	±	0.8	4.4	±	0.7
7	$4._{2}$	±	$1._{0}$	$4._{1}$	±	$1._{0}$
8	$3._{7}$	±	$1._{2}$	4.2	±	0.7
9	$4._{1}$	±	$1._{0}$	4.5	±	0.8
10	4.1	±	0.9	4.4	±	0.5
11	$3._{7}$	±	$1._{0}$	4.5	±	0.5
12	4.4	±	0.7	4.5	±	0.8

[a]Values are the average of 5-point Likert scale responses to questions in Table S-2 of 11 REU peers (self evaluations were excluded)

**Table S6.** REU Assessment of REU Peers[a] Question 3: experimental data support findings						
	Week 2			Week 10		
Subject	Ave		SD	Ave	±	SD
1	4.5	±	0.7	4.5	±	0.8
2	3.8	±	0.9	4.4	±	0.8
3	3.5	±	0.8	4.4	±	0.5
4	4.3	±	0.6	4.5	±	0.5
5	4.4	±	0.7	4.5	±	0.5
6	$3._{6}$	±	$1._{1}$	4.5	±	0.5
7	$3._{6}$	±	$1._{0}$	4.4	±	0.7
8	$3._{9}$	±	$1._{0}$	3.8	±	0.8
9	4.2	±	0.9	4.6	±	0.5
10	4.2	±	0.8	4.3	±	0.8
11	4.0	±	0.9	4.2	±	0.9
12	4.5	±	0.7	4.6	±	0.5

[a]Values are the average of 5-point Likert scale responses to questions in Table S-2 of 11 REU peers (self evaluations were excluded)

Qualitative Assessment of Weekly Research Reports.

Each weekly research report recorded via GoTo Meeting was transcribed word for word and each student researcher's transcripts were connected together in consecutive order. Transcripts were analyzed using a closed coding system looking specifically for language indicating ownership of the research ideas, research tasks, and research findings. Once the transcripts were coded, the transcripts were grouped based on emerging trends in language usage. Each of the 2017 REU participants demonstrated ownership of the presentations, with all referring to themselves (e.g. "as I've shown here", "as I've stated previously", "my graph here shows…") when speaking about something specifically related to the presentation. This indicates that each student created his or her own slides and maintains ownership of the presentations. Further qualitative assessment was performed to document the degree to which each particpant took individual ownership of the project, goals, or findings. Three groupings emerged in terms of how students utlize language to describe their research each week.

The first group showed a high degree of ownership of their project as well as recognition of their role in a larger group goal. However, since their degree of ownership started out very high, there was little evidence of increased ownership throughout the duration of the project. These students (n=3) referred to their research using first person language and described their research in such a way that it was their own project. For instance, Participant 11 began each presentation by stating, "this week I was working on…" indicating recognition of being part of a larger group in which the student's individual work played a role in the outcome. Participant 11 goes on to describe "my goal for this week…" and "my findings…" for the week. Similarly, Participants 3 and 9 also used singular first person language to discuss their research goals, and findings. However, during the results reporting, Participants 3 and 9 also used some plural first person language. For instance participant 9 indicated that "in the past few weeks we've had some setbacks" when describing a change in the direction of the research and that with those changes "we can look for inconsistencies later."

The second group (n=4) is comprised of student researchers who showed movement in their level of ownership of the research project. There were two subgroups within this group, one subgroup (n=3) showed an increase in individual ownership and the second (n=1) showed a decrease in individual ownership of the project. For instance, Participant 12 began the first week's presentation by describing that "this past week we continued our project." This participant goes on to describe "the goals for this past week" and what "the findings" included. The specific use of "the goals" and "the findings" indicates very weak ownership of the project. However, by week 4 (of 10), the participant transitions to using language that is more indicative of group ownership. For instance, the participant describes "our goals for this week" and goes on to discuss "our findings for this week". By the week six presentation, indicators of more ownership progress as evidenced by the participant describing the work that was done "in my project this week". However, use of language indicative of group ownership continued throughout the rest of the presentations. Participants 6 and 8 show similar increases of ownership in their research. However, each participant began at a different level of ownership. Participant 6 began each presentation by describing the project "we are working on". While this particpant identified "my goals" for the week as being distinctly related to the work in process, discussion about "the findings" was made with little or no ownership. However, by the week four presentation, the level of ownership by Participant 6 increased as evidenced by the description of the project "I'm working on" this week as well as individual ownership of "my goals for this last week" as well as reporting on "my findings". While describing the results of the week's work, this particpant often described the presentation slides by indicating group ownership. During week four, Participant 6 indicated that "up here we have images…". However, by week eight and nine, the use of plural references reduced and there is a transition to taking more individual

ownership by describing the findings in the following manner, “I also found that...”. Participant 8 began the first week’s presentation by talking about “my project”, which does indicate ownership. However, Participant 8 goes on to describe “our goals” and “our findings” for the week. The majority of the language used to describe the findings indicates plural ownership (e.g. we, us). By week six, Participant 8 utilizes language related to more indivdual ownership (i.e. “my project”, “my goals”, “my findings”), but continues through the rest of the weekly presentations to utilze the plural ownership when describing the actual results.

The fourth participant in this group (Participant 1), showed a transition as well, but in this case the transition was from more individual ownership to more of a group ownership within the research group. Particpant 1 began the first presentation by discussing “the goals for last week” indicating no language of ownership and talking about “my findings” indicating ownership of the work accomplished by this individual. By week four, Participant 1 was referring the both the goals and the findings in such a way as to indicate individual ownership (i.e. “my goals”, “my major findings”), while continuing to utilize plural ownership when discussing the results. By the week 10 presentation, Participant 1 was utlizing all plural ownership language by discussing “our goals for this past week” and “our findings for the week”.

The third and final group showed little or no change in their sense of ownership through this analysis and began with relatively low ownership of the research. Since all participants in this group (n=5) utilized similar language conventions, discussion will be limited to a few representative examples. While each participant indicated some sense of ownership of the research, most of the ownership was at the group level. For instance, Participant 10 started each presentation with the phrase “I will be discussing…” or something similar. Each of the researchers in this group described “our goals for this past week”, (Participant 5, Week 6 presentation) indicating a sense of belonging to the research group but lack of individual ownership of the tasks at hand for the week. Similarly, participants in this group showed a lack of individual ownership of the findings of their work from the previous week. For example, Participant 7 talks about what “our data had shown” (Week 10) for the prior week and Participant 2 describes what “our findings were” (Week 9). All participants in this group utilized predominantly language indicating group ownership (e.g. we, our) when discussing the results.
